# Camlipixant in Refractory Chronic Cough: A Phase 2a, Randomized Controlled Trial (RELIEF)

**DOI:** 10.1164/rccm.202501-0093RL

**Published:** 2025-03-05

**Authors:** Jaclyn A. Smith, Alyn H. Morice, Surinder S. Birring, Sean M. Parker, Paul A. Marsden, John R. Holcomb, Mandel Sher, Bruce M. Prenner, Gary Steven, Kevin J. Carroll, Sylvain Lanouette, Denis Garceau, Laurent Harvey, Catherine M. Bonuccelli

**Affiliations:** ^1^ Division of Immunology, Immunity to Infection and Respiratory Medicine, University of Manchester and Manchester University NHS Foundation Trust, Manchester, United Kingdom;; ^2^ Respiratory Medicine, Hull York Medical School, University of Hull, Hull, United Kingdom;; ^3^ Centre for Human and Applied Physiological Sciences, School of Basic and Medical Biosciences, King’s College London, London, United Kingdom;; ^4^ Department of Respiratory Medicine, Northumbria Healthcare NHS Foundation Trust, North Shields, United Kingdom;; ^5^ Diagnostics Research Group, San Antonio, Texas;; ^6^ Center for Cough, Largo, Florida;; ^7^ Allergy Associates Medical Group, San Diego, California;; ^8^ Allergy, Asthma & Sinus Center, Milwaukee, Wisconsin;; ^9^ KJC Statistics Ireland, Dublin, Ireland;; ^10^ Bellus Health, Montreal, Quebec, Canada; and; ^11^ Bellus Health, Wilmington, Delaware

*To the Editor*:

Chronic cough, lasting >8 weeks, has an estimated global prevalence of 10%, variable by region (range, 2–18%) (1, 2). Chronic cough is associated with morbidities including sleep disturbance, fatigue, urinary incontinence, and depression (2).

Refractory chronic cough (RCC), with no identifiable cause despite adequate investigation or unresponsive to optimal treatment of associated conditions, represents a significant proportion of chronic cough cases (3, 4). Treatment options for RCC are typically limited; off-label options may have suboptimal efficacy, poor tolerability, or raise concerns about the risk of addiction (4–6).

Clinical trials of a first-generation P2X3 antagonist in patients with RCC resulted in improvements in cough and quality of life (7), but highlighted significant incidence of taste disturbance (8). Camlipixant, another P2X3 antagonist, was well tolerated in healthy volunteers, with an acceptable safety profile and no complete taste loss (ageusia) at any dose (9). Here we report the results of RELIEF (NCT03979638), a multicenter (United Kingdom, eight; United States, eight), randomized, double-blind, placebo-controlled, dose-escalation phase 2a trial with a two-period crossover that assessed safety and efficacy of escalating camlipixant doses in patients with RCC.

**Figure 1.**
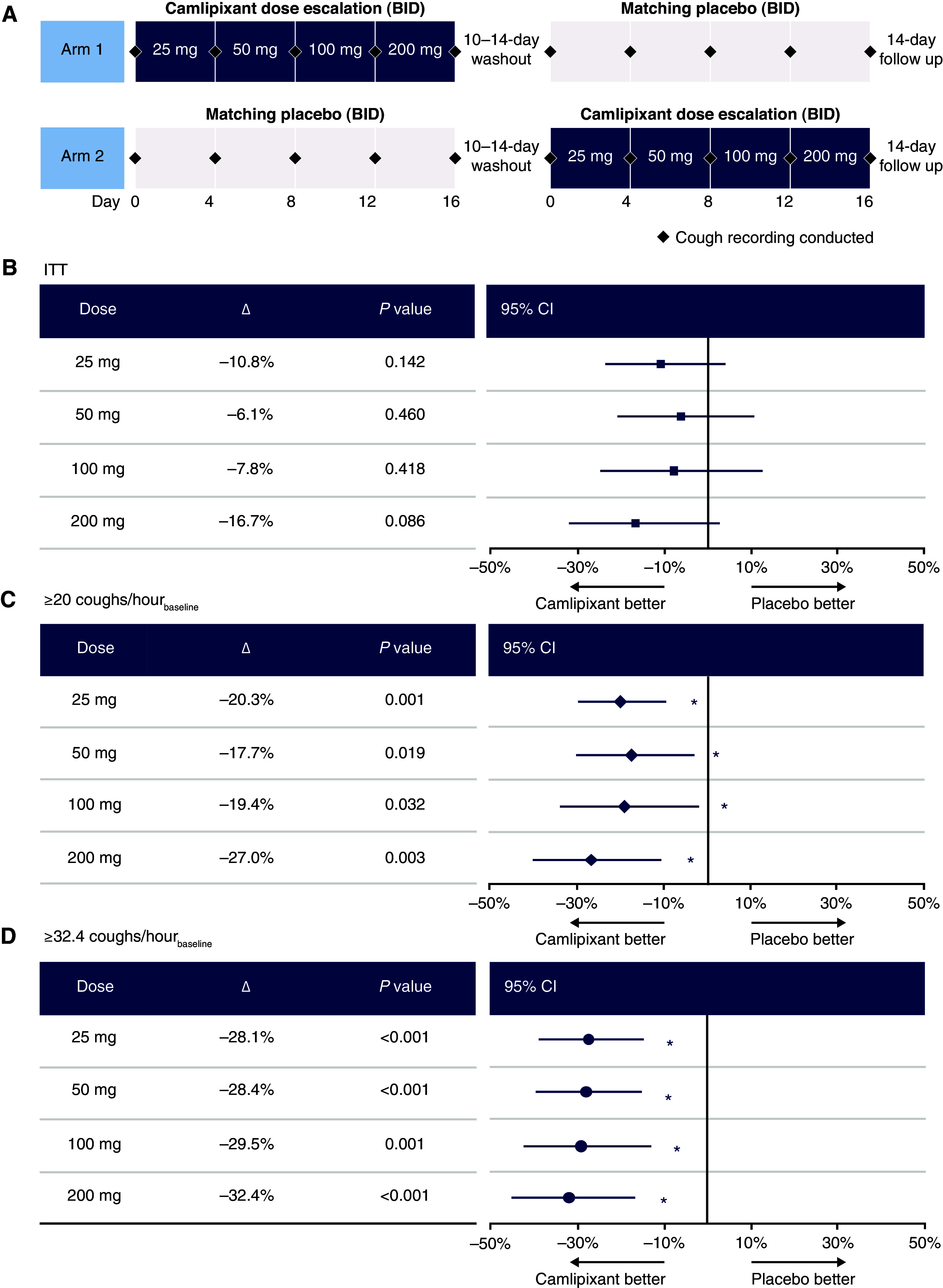
(*A*) Study design and (*B–D*) placebo-adjusted change from baseline in awake cough frequency. Forest plot of mean placebo-adjusted change in awake cough frequency for (*B*) the intention-to-treat (ITT) population and (*C* and *D*) prespecified subgroups at each dose level. Error bars represent 95% CI. *P* values (two-sided) are presented. Patients were treated BID for 4 days at each dose level. *P < 0.05. A mixed model for repeated measures was used to compare camlipixant with placebo for all cough frequency endpoints; data were analyzed on a natural log scale. Sensitivity analyses assessed the primary efficacy endpoint in the per-protocol and completers groups, through worst-case imputation, and in the ITT population. BID = twice daily; CI = confidence interval.

Eligible patients were aged 18–80 years, had RCC for ⩾1 year, and had an awake cough frequency ⩾10 coughs/h at screening, measured by ambulatory cough recording. Patients were randomized 1:1 to receive camlipixant or placebo for 16 days (Figure 1A). After a 10- to 14-day washout period, patients crossed over to the alternative 16-day treatment period. Escalating camlipixant doses were orally administered (25, 50, 100, and 200 mg, all twice daily), each over 4 days. Matched placebo tablets were administered twice daily during the placebo treatment period.

The primary efficacy endpoint was placebo-adjusted change from baseline in awake cough frequency at each dose. Prespecified analyses also investigated the interaction between baseline awake cough frequency and treatment effect. Prespecified analyses included efficacy assessment in the intent-to-treat (ITT) population and two additional subgroups, defined by baseline awake cough frequency of >20 coughs/h or ⩾20 coughs/h, and < median cough frequency or ⩾ median cough frequency. Secondary efficacy endpoints included change in 24-hour cough frequency, cough severity visual analog scale, and Leicester Cough Questionnaire (LCQ). Safety outcomes included the incidence of adverse events (AEs).

It was estimated that 62 randomized patients would provide 80% power, assuming a true reduction of 32% in awake cough frequency with camlipixant versus placebo using a two-tailed 5% overall significance level (10). Recruitment of 68 patients was planned to account for dropouts. No adjustments were made for multiplicity.

All patients signed an informed consent form before screening. Detailed methods, including ethical statements, and results are in the online supplement.

Overall, 107 patients were assessed for eligibility. The safety analysis population comprised 68 patients who received either treatment sequence; 67 patients were included in the ITT population. At premature trial termination due to the coronavirus disease (COVID-19) pandemic, 52 patients had completed both dosing periods.

In the safety population (*N* = 68), the mean age of patients was 64.0 (SD, 10.5) years (range, 21–80 yr), and 85% were female. Mean cough duration was 14.7 (SD, 9.9) years. The median baseline awake cough frequency was 32.4 coughs/h (range, 10–183 coughs/h). Baseline characteristics were similar between arms.

Numerical reductions in awake cough frequency from baseline were observed with camlipixant at all doses versus placebo in the ITT population, but these were not statistically significant (Table 1). At all doses of camlipixant, placebo-adjusted changes from baseline in awake cough frequency were statistically significant in subgroups with ⩾20 and with ⩾32.4 coughs/h (median value) at baseline. A similar trend was observed with 24-hour cough frequency. A significant interaction between baseline awake cough frequency and primary efficacy outcome was confirmed across doses in the ITT population (*P* = 0.026), suggesting the magnitude of the treatment effect increased with baseline cough frequency.

**Table 1.** Awake Cough Frequency

	Day 0 Baseline	Day 4, 25 mg Twice Daily	Day 8, 50 mg Twice Daily	Day 12, 100 mg Twice Daily	Day 16, 200 mg Twice Daily	Washout/Follow-Up
ITT						
Camlipixant, *n*	61	60	59	56	58	51
Arithmetic mean (SD)	37.4 (30.7)	31.5 (24.0)	29.5 (20.3)	30.5 (23.7)	29.1 (22.4)	39.0 (29.4)
Geometric mean	28.0	24.3	23.6	23.5	21.9	30.3
Geometric mean ratio vs. baseline* (95% CI)	—	0.9 (0.8 to 1.0)	0.9 (0.7 to 1.0)	0.8 (0.7 to 1.0)	0.8 (0.6 to 0.9)	1.0 (0.9 to 1.2)
Placebo, *n*	60	59	58	58	58	52
Arithmetic mean (SD)	42.6 (35.8)	40.0 (33.4)	38.3 (28.4)	39.8 (35.9)	39.3 (32.9)	36.6 (33.5)
Geometric mean	32.1	28.3	27.5	27.0	27.0	26.7
Geometric mean ratio vs. baseline* (95% CI)	—	0.9 (0.8 to 1.0)	0.8 (0.7 to 0.9)	0.8 (0.7 to 0.9)	0.8 (0.7 to 0.9)	0.8 (0.7 to 0.9)
Placebo-adjusted change from baseline,**^†^** % (95% CI)	—	−10.8 (−23.5 to 4.0)	−6.1 (−20.8 to 11.2)	−7.8 (−24.4 to 12.5)	−16.7 (−32.3 to 2.6)	—
*P* value	—	0.142	0.460	0.418	0.086	—
Subgroup of patients with ⩾20.0 coughs/h_baseline_^‡^						
Camlipixant, *n*	47	46	45	44	45	40
Arithmetic mean (SD)	45.1 (31.0)	37.6 (24.3)	34.6 (20.5)	35.2 (24.6)	33.9 (23.1)	45.0 (29.8)
Geometric mean	37.3	31.3	29.3	28.3	26.8	37.5
Geometric mean ratio vs. baseline* (95% CI)	—	0.8 (0.7 to 0.9)	0.8 (0.7 to 0.9)	0.8 (0.6 to 1.0)	0.7 (0.6 to 0.9)	0.9 (0.8 to 1.1)
Placebo, *n*	47	46	46	47	46	41
Arithmetic mean (SD)	50.9 (36.3)	48.6 (33.0)	45.7 (27.3)	46.9 (36.3)	46.9 (32.9)	43.6 (34.5)
Geometric mean	41.9	39.9	37.5	35.9	36.7	34.8
Geometric mean ratio vs. baseline* (95% CI)	—	1.0 (0.9 to 1.1)	0.9 (0.8 to 1.0)	0.9 (0.7 to 1.0)	0.9 (0.7 to 1.0)	0.8 (0.7 to 1.0)
Placebo-adjusted change from baseline,^†^ % (95% CI)	—	−20.3 (−29.9 to −9.5)	−17.7 (−29.9 to −3.3)	−19.4 (−33.9 to −1.9)	−27.0 (−40.3 to −10.8)	—
* P* value	—	0.001	0.019	0.032	0.003	—
Subgroup of patients with ⩾32.4 coughs/h_baseline_						
Camlipixant, *n*	28	28	28	27	28	26
Arithmetic mean (SD)	59.1 (32.7)	48.2 (25.0)	43.2 (20.8)	43.3 (26.3)	42.2 (23.2)	55.6 (32.1)
Geometric mean	52.5	42.6	38.4	36.8	35.5	46.9
Geometric mean ratio vs. baseline* (95% CI)	—	0.8 (0.7 to 0.9)	0.7 (0.6 to 0.9)	0.7 (0.6 to 0.9)	0.7 (0.6 to 0.8)	0.9 (0.7 to 1.1)
Placebo, *n*	31	30	31	31	31	26
Arithmetic mean (SD)	65.0 (37.3)	61.9 (33.8)	56.0 (26.5)	59.6 (38.5)	56.5 (33.2)	54.6 (38.6)
Geometric mean	57.4	53.8	49.3	49.0	48.2	45.5
Geometric mean ratio vs. baseline* (95% CI)	—	0.9 (0.8 to 1.1)	0.9 (0.8 to 1.0)	0.9 (0.7 to 1.0)	0.8 (0.7 to 1.0)	0.8 (0.7 to 0.9)
Placebo-adjusted change from baseline,^†^ % (95% CI)	—	−28.1 (−39.5 to −14.5)	−28.4 (−39.7 to −15.0)	−29.5 (−42.7 to −13.2)	−32.4 (−45.3 to −16.4)	—
* P* value	—	<0.001	<0.001	0.001	<0.001	—

*Definition of abbreviations*: CI = confidence interval; ITT = intent-to-treat.

* Calculated as least-square mean change from baseline on the log-scale from the fitted model.

^†^ The placebo-adjusted percentage change from baseline was estimated as 100(*e^difference^* − 1), where the difference represents the geometric mean ratio, on the natural log scale.

^‡^ Overall, patients with ⩾20 awake coughs/h represented 79% of the ITT population.

Placebo-adjusted changes in cough severity visual analog scale from baseline in patients treated with camlipixant after 16 days were −4.6 mm (95% confidence interval [CI], −12.5 to 3.3) in the ITT population, −7.6 mm (95% CI, −16.3 to 1.1) in the ⩾20.0 coughs/h subgroup, and −11.5 mm (95% CI, −22.9 to −0.2) in the ⩾32.4 coughs/h subgroup.

Improvements in mean LCQ scores from baseline in patients treated with camlipixant after 16 days were 1.8, 2.3, and 2.6 for the ITT population, ⩾20.0, and ⩾32.4 coughs/h subgroups, respectively. In contrast, average improvements of 1.1, 1.0, and 1.3 from baseline LCQ scores were reported with placebo for the ITT population, ⩾20.0, and ⩾32.4 coughs/h subgroups, respectively.

Similar cumulative incidences of treatment-emergent AEs were reported for camlipixant (68.9% of patients) and placebo (67.2% of patients), and proportions of patients with treatment-emergent AEs did not increase with camlipixant dose. Most treatment-emergent AEs were mild to moderate in severity. Four severe treatment-emergent AEs were reported in two patients. Investigators did not consider any severe AEs to be treatment-related. No serious AEs were reported during the trial. Overall, treatment-emergent taste disturbance AEs (dysgeusia, hypogeusia, or ageusia) were reported in six patients receiving camlipixant (9.8%) and three patients receiving placebo (4.9%). No patients reported ageusia or discontinued study treatment because of taste disturbance.

In conclusion, the primary endpoint of change from baseline in awake cough frequency did not reach statistical significance at any camlipixant dose in the ITT population; however, this was achieved at all doses in predefined patient subgroups with baseline awake cough frequency ⩾20 or ⩾32.4 coughs/h. There were no indications of a direct relationship between camlipixant dose and reductions in awake cough frequency.

Trial limitations included the study design being fixed-sequence, short-duration, within-patient dose escalation without a washout between doses, which did not allow confirmatory characterization of dose–response. This trial was not powered to observe a difference versus placebo in patient-reported outcomes, and each dosing period was short (4 d). Nonetheless, patient-reported outcomes included numerical improvements in perceived cough severity and cough-specific quality of life with camlipixant versus placebo.

Although the primary endpoint was not met, these data suggest that camlipixant may be an effective and well-tolerated therapy across the dose range studied in patients with RCC, consistent with the subsequent phase 2b SOOTHE trial (NCT04678206) (11).
